# 337. SARS-CoV-2 Viral Load Does Not Predict Incident Venous Thromboembolism in COVID-19

**DOI:** 10.1093/ofid/ofab466.538

**Published:** 2021-12-04

**Authors:** Simon Pollett, Benjamin Wier, Stephanie A Richard, Anthony C Fries, Ryan C Maves, Ryan C Maves, Gregory Utz, Tahaniyat Lalani, Rupal Mody, Anuradha Ganesan, Rhonda E Colombo, Chris Colombo, David A Lindholm, David A Lindholm, Cristian Madar, Sharon Chi, Nikhil Huprikar, Derek Larson, Samantha Bazan, Ann Scher, Jennifer Rusiecki, Celia Byrne, Katrin Mende, Mark P Simons, David Tribble, Brian Agan, Timothy Burgess

**Affiliations:** 1 Uniformed Services University of the Health Sciences, Bethesda, Maryland; 2 Infectious Disease Clinical Research Program, Department of Preventive Medicine and Biostatistics, Uniformed Services University of the Health Sciences, Bethesda, MD and Henry M. Jackson Foundation, Bethesda, MD, Bethesda, Maryland; 3 United States Air Force School of Aerospace Medicine, Wright-Patterson AFB, Ohio; 4 Naval Medical Center San Diego, San Diego, CA and Infectious Disease Clinical Research Program, Bethesda, MD, San DIego, California; 5 IDCRP, HJF, and NMCP, Bethesda, Maryland; 6 WBAMC, El Paso, Texas; 7 Infectious Disease Clinical Research Program and the Henry M. Jackson Foundation for the Advancement of Military Medicine and Walter Reed National Military Medical Center, Bethesda, MD; 8 Madigan Army Medical Center, Tacoma, WA, Infectious Disease Clinical Research Program, Bethesda, MD, and Henry M. Jackson Foundation for the Advancement of Military Medicine, Inc., Bethesda, MD, Tacoma, Washington; 9 Madigan Army Medical Center, Joint Base Lewis-McChord, Washington; 10 Uniformed Services University of the Health Sciences; Brooke Army Medical Center, San Antonio, TX; 11 Tripler Army Medical Center, Tripler Army Medical Center, Hawaii; 12 Infectious Disease Clinical Research Program, Bethesda, Maryland; 13 Walter Reed National Military Medical Center (WRNMMC), Bethesda, Maryland; 14 Fort Belvoir Community Hospital Infectious Disease, Fort Belvoir, Virginia; 15 Carl R. Darnall Army Medical Center, Fort Hood, Texas; 16 USUHS, Bethesda, Maryland; 17 Infectious Disease Clinical Research Program, Bethesda, MD, The Henry M. Jackson Foundation, Bethesda, MD, and Brooke Army Medical Center, Fort Sam Houston, TX, San Antonio, TX; 18 Infectious Disease Clinical Research Program, USU/HJF, Bethesda, Maryland

## Abstract

**Background:**

The risk factors of venous thromboembolism (VTE) in COVID-19 warrant further study. We leveraged a cohort in the Military Health System (MHS) to identify clinical and virological predictors of incident deep venous thrombosis (DVT), pulmonary embolism (PE), and other VTE within 90-days after COVID-19 onset.

**Methods:**

PCR or serologically-confirmed SARS-CoV-2 infected MHS beneficiaries were enrolled via nine military treatment facilities (MTF) through April 2021. Case characteristics were derived from interview and review of the electronic medical record (EMR) through one-year follow-up in outpatients and inpatients. qPCR was performed on upper respiratory swab specimens collected post-enrollment to estimate SARS-CoV-2 viral load. The frequency of incident DVT, PE, or other VTE by 90-days post-COVID-19 onset were ascertained by ICD-10 code. Correlates of 90-day VTE were determined through multivariate logistic regression, including age and sampling-time-adjusted log10-SARS-CoV-2 GE/reaction as *a priori* predictors in addition to other demographic and clinical covariates which were selected through stepwise regression.

**Results:**

1473 participants with SARS-CoV-2 infection were enrolled through April 2021. 21% of study participants were inpatients; the mean age was 41 years (SD = 17.0 years). The median Charlson Comorbidity Index score was 0 (IQR = 0 - 1, range = 0 - 13). 27 (1.8%) had a prior history of VTE. Mean maximum viral load observed was 1.65 x 10^7^ genome equivalents/reaction. 36 (2.4%) of all SARS-CoV-2 cases (including inpatients and outpatients), 29 (9.5%) of COVID-19 inpatients, and 7 (0.6%) of outpatients received an ICD-10 diagnosis of any VTE within 90 days after COVID-19 onset. Logistic regression identified hospitalization (aOR = 11.1, p = 0.003) and prior VTE (aOR = 6.2 , p = 0.009) as independent predictors of VTE within 90 days of symptom onset. Neither age (aOR = 1.0, p = 0.50), other demographic covariates, other comorbidities, nor SARS-CoV-2 viral load (aOR = 1.1, p = 0.60) were associated with 90-day VTE.

**Conclusion:**

VTE was relatively frequent in this MHS cohort. SARS-CoV-2 viral load did not increase the odds of 90-day VTE. Rather, being hospitalized for SARS-CoV-2 and prior VTE history remained the strongest predictors of this complication.

**Disclosures:**

**Simon Pollett, MBBS**, **Astra Zeneca** (Other Financial or Material Support, HJF, in support of USU IDCRP, funded under a CRADA to augment the conduct of an unrelated Phase III COVID-19 vaccine trial sponsored by AstraZeneca as part of USG response (unrelated work)) **Ryan C. Maves, MD**, **EMD Serono** (Advisor or Review Panel member)**Heron Therapeutics** (Advisor or Review Panel member) **David A. Lindholm, MD**, American Board of Internal Medicine (Individual(s) Involved: Self): Member of Auxiliary R&D Infectious Disease Item-Writer Task Force. No financial support received. No exam questions will be disclosed ., Other Financial or Material Support **David Tribble, M.D., DrPH**, **Astra Zeneca** (Other Financial or Material Support, HJF, in support of USU IDCRP, funded under a CRADA to augment the conduct of an unrelated Phase III COVID-19 vaccine trial sponsored by AstraZeneca as part of USG response (unrelated work))

